# Epidemiology, transmission, vector and factors analysis of chikungunya outbreak in 2025, Quanzhou City of China

**DOI:** 10.3389/fpubh.2026.1895402

**Published:** 2026-07-28

**Authors:** Shenggen Wu, Jiayuan Xie, Hongfeng Zhao, Jiangyi Liu, Ruyi Guo, Senshuang Zheng, Jiawei Lin, Jingjing Cai, Wenjing Ye, Qiuping Chen, Roger Frutos, Yanhua Su, Jianming Ou, Weiming Wang, Yuwei Weng

**Affiliations:** 1 Fujian Center for Disease Control and Prevention, Fujian Provincial Key Laboratory of Zoonosis Research, Fuzhou, Fujian, China; 2 School of Public Health, Fujian Medical University, Fuzhou, China; 3 State Key Laboratory of Vaccines for Infectious Diseases, Xiang An Biomedicine Laboratory, State Key Laboratory of Molecular Vaccinology and Molecular Diagnostics, National Innovation Platform for Industry-Education Integration in Vaccine Research, School of Public Health, Xiamen University, Xiamen, Fujian, China; 4 Quanzhou Center for Disease Control and Prevention, Quanzhou, China; 5 Department of Infectious Diseases, Quanzhou First Hospital Affiliated to Fujian Medical University, Quanzhou, China; 6 CIRAD, URM 17, Intertryp, Montpellier, France; 7 Department of Pathology, Faculty of Medicine-Ramathibodi Hospital, Mahidol University, Bangkok, Thailand; 8 Department of Health, Faculty of Vocational Studies, Universitas Airlangga, Surabaya, Indonesia

**Keywords:** chikungunya fever, cycle threshold value, epidemiology, outbreak, risk factors

## Abstract

**Background:**

Following a major chikungunya outbreak in Foshan City, China, locally transmitted cases have been reported across the country. Research remains limited to epidemiology, transmission dynamics, vector characteristics, and risk factors of chikungunya fever.

**Methods:**

We analyzed 164 chikungunya cases reported in Quanzhou City, Fujian Province, between August 18 and September 9, 2025, along with mosquito surveillance data collected from August 25 to September 13, 2025. We examined case characteristics, symptom patterns, Cycle threshold (Ct) values trends, and vector distributions. Logistic regression was used to identify demographic factors and mosquito density indices associated with infection risk.

**Results:**

Among 164 cases, 50.0% were male and 38.4% were aged ≥ 60 years. Ct values were significantly influenced by age (*p* = 0.0044) and the onset-to-diagnosis intervals (*p* < 0.0001), but not by symptom patterns. Advanced age was a major risk factor, particularly for individuals aged 70–89 years (OR = 6.18, 95% CI = 4.08–8.98) and those 90 years or older (OR = 4.37, 95% CI = 1.36–10.29). Higher mosquito larval and adult densities also increased infection risk. Following implementation of emergency response protocols and intensive mosquito control measures, zero incident cases were reported within 21 days.

**Conclusion:**

Chikungunya disproportionately affects older adults, especially those aged ≥ 70 years. Viral load increases with age and decreases with delayed diagnosis. The lack of correlation between symptoms and viral load may suggest that asymptomatic individuals could be equally infectious, providing guidance for the screening and management of high-risk populations.

## 1 Introduction

Chikungunya fever is an acute vector-borne infectious disease caused by the chikungunya virus (CHIKV), which has shown rapid spread and outbreak epidemics worldwide in recent years. Local transmission has been reported in 119 nations and areas (1, 2). Climate change and increased international travel have heightened the risk of transmission in non-endemic areas, establishing chikungunya fever as a major global public health threat (3, 4). In China, most cases have historically been imported, though the risk of local transmission has increased substantially in recent years. The first locally transmitted outbreak in China occurred in Guangdong province in 2010 (5). Imported cases rose steadily from 2015 to 2019 but declined sharply between 2020 and 2022 due to COVID-19 prevention measures (6). In July 2025, Foshan City in Guangdong Province experienced the largest local outbreak since 2010 (7). Although this outbreak has been contained, China’s extensive *Aedes* mosquito distribution and general lack of population immunity create vector circumstances that are favorable for local CHIKV transmission. Future local epidemics brought on by imported patients are still a possibility.

Epidemiological evidence on chikungunya fever outbreak risk remains limited. Reviews have identified key knowledge gaps, including understanding of high-risk populations, outbreak scale, and the duration of natural immunity (8, 9). While several studies suggest that infection risk may vary according to individual characteristics (10–12), more evidence is needed to better understand these patterns and inform targeted prevention strategies.

Few studies have examined the relationship between cycle threshold (Ct) values and clinical symptoms or patient characteristics. A study of the 2016 outbreak in Delhi, India, discovered correlations between various clinical characteristics and CHIKV burden (13). Research on the 2025 Foshan, China outbreak demonstrated that viral loads varied according to peak infection periods and symptom onset-to-diagnosis intervals (14). However, the complex relationships among viral load, patient age, time to diagnosis, and clinical presentation remain unclear.

Additionally, results on the effect of interventions on epidemic curves and the influence of vector surveillance measures like the Adult Density Index (ADI) and Breteau Index (BI) on chikungunya epidemics are still conflicting. Compared to dengue, research on vector control for chikungunya is far less common. While reviews have shown that environmental interventions can reduce vector indices, the quality of evidence linking these reductions to decreased disease incidence is poor (15–17). Though chikungunya and dengue share the same Aedes mosquito vectors and control strategies may apply to both diseases, further research is needed to confirm the effectiveness of mosquito control measures specifically for Chikungunya.

Thus, this study described the epidemiological characteristics of the 2025 chikungunya outbreak in Quanzhou City, Fujian Province. We aim to examine how Ct values relate to age, onset-to-diagnosis interval and clinical presentation, and identify risk factors associated with infection to inform evidence-based prevention and control strategies.

## 2 Materials and methods

### 2.1 Data sources

164 case data in Quanzhou from August 18 to September 9 were obtained. Sex, age, occupation, current address (district/street), date of onset of disease, date of diagnosis, Ct value of the first positive test, and symptoms were among the dataset. Detailed symptom information was additionally retrieved from hospital records via retrospective questionnaires. As one case was asymptomatic and four cases did not complete the questionnaire due to recall difficulty over time, a total of 159 cases were ultimately included and the small proportion excluded is unlikely to have biased the subsequent symptom analyses.

Mosquito surveillance data from 328 sampling sites of Quanzhou between August 25 and September 13 was acquired. These data were obtained through special entomological surveys and routine vector monitoring systems implemented during the outbreak. At the community (administrative village) level, daily emergency surveillance was conducted using the BI method for larval monitoring and the double-layered tent trapping method for adult mosquito monitoring, with the intensity and coverage adjusted based on outbreak dynamics and manpower. The larval density index is: BI = (number of positive containers ÷ number of households surveyed) × 100, where positive containers refer to waterlogged containers where *Aedes aegypti* larvae (tsetse) or pupae are detected by inspection. One household is defined as every 30 square meters of area in residential homes, collective dormitories (with two rooms counted as one household), or public spaces. The BI values below 5 represents the threshold for controlling mosquito-borne disease transmission, 5 to 10 indicates transmission risk, 10 to 20 indicates risk of clustered outbreaks, and above 20 indicates risk of localized outbreaks. ADI: Tent Lure Index (only/top-hour = number of female *Aedes aegypti* mosquitoes captured (only)/duration (minutes) × 60 min).

### 2.2 Case definition

According to the *Diagnosis of Chikungunya Fever* (WS/T 590–2018) issued by the National Health Commission of the People’s Republic of China (18), confirmed cases were defined as suspected or clinically diagnosed cases meeting at least one of the following laboratory criteria: (1) Detection of chikungunya virus RNA by nucleic acid amplification methods such as RT-PCR; (2) Isolation of chikungunya virus from the patient’s serum; (3) Either a four-fold or greater increase in serum chikungunya virus-specific IgG antibody titer from the acute to the recovery phase, or detection of chikungunya virus-specific IgM antibody in acute-phase serum. In this study, all 164 confirmed chikungunya cases tested positive by RT-PCR and yielded valid Ct values, with all assays performed using a single standardized protocol at one institutional laboratory. Serological criteria were used only as supplementary confirmation.

### 2.3 Statistical analysis

We analyzed the epidemiological characteristics of the outbreak by examining demographic characteristics and tempo-spatial distributions of reported cases. Demographic variables (age, sex, occupation) and region were compared between groups using Fisher’s exact test. Cases were further characterized by onset-to-diagnosis intervals, clinical symptoms, Ct values, and BI and ADI distributions. Spearman’s rank correlation was used to examine the association between BI/ADI and the number of reported cases. Data visualization was performed using Python 3.7, and statistical analyses were conducted using SPSS 24.0. All tests were two-sided, with *p* < 0.05 considered statistically significant.

Chi-square tests were used to compare categorical variables. We applied the Kruskal-Wallis test for general comparisons and Bonferroni-corrected Dunn post-hoc tests for pairwise comparisons to evaluate the association between Ct values and several parameters. Multiple linear regression analyses were performed, with Ct values as the dependent variable and sex, age, occupation, district, onset-to-diagnosis interval, and symptoms as the independent variables. Variables were coded as stated in Table 1.

**Table 1:** Variable coding scheme for multiple linear regression analysis.

Variable	Coding
Sex	0 = Male, 1 = Female
Age(years)	0 = 0–9, 1 = 10–29, 2 = 30–49, 3 = 50–69, 4 = 70–89, 5 = ≥ 90
Occupation	0 = Student, 1 = Commercial/service, 2 = Farmer, 3 = Retiree, 4 = Teacher, 5 = Homemaker/unemployed, 6 = Civil servant, 7 = Office worker, 8 = Manual worker, 9 = Self-employed
District	0 = Licheng, 1 = Luojiang, 2 = Fengze, 3 = Jinjiang, 4 = Nan’an
Symptom onset-to-diagnosis interval (days)	0 = 0, 1 = 1, 2 = 2, 3 = 3, 4 = 4, 5 = 5, 6 = 6, 7 = 7
Symptoms	1 = Fever, 2 = Fever and arthralgia, 3 = Fever and rash, 4 = Fever, rash, and arthralgia, 5 = Arthralgia, 6 = Arthralgia and fever, 7 = Arthralgia and rash, 8 = Rash, 9 = Asymptomatic

To further investigate the relationship between Ct values and onset-to-diagnosis interval, we fitted Ct values using nonlinear least squares regression with the following function [Equation 1] (19):


$$y=b_{1}-b_{2}x^{-0.5}+b_{3}x^{-1}$$ (1)

To identify factors associated with chikungunya fever risk, we conducted logistic regression analysis using the LogisticRegression module of the scikit-learn package in Python 3.7. The model assessed associations between demographic factors (age, sex), mosquito surveillance indices (BI, ADI), and infection risk, with each daily confirmed case treated as an independent unit of analysis. BI and ADI values were assigned according to the monitoring period and site corresponding to each reported case, and cases from the same surveillance unit therefore shared identical exposure levels. The regression model was specified as follows [Equation 2]:


$$\text{logit}(P)=\ln(\frac{P}{1-P})=\beta_{0}+\beta_{1}X_{1}+\beta_{2}X_{2}+\ldots +\beta_{n}X_{n}$$ (2)

*P* represents the probability of infection in the study population; X₁, X₂,., X_n_ are the independent variables (age, sex, BI, ADI); *β₀* is the intercept representing the log-odds of infection when all independent variables equal zero; and *β₁*, *β₂*,., *β*_n_ are the regression coefficients for the respective variables.

## 3 Results

### 3.1 Epidemiological characteristics of chikungunya fever

#### 3.1.1 Demographic, temporal and spatial distribution

**Figure 1:**
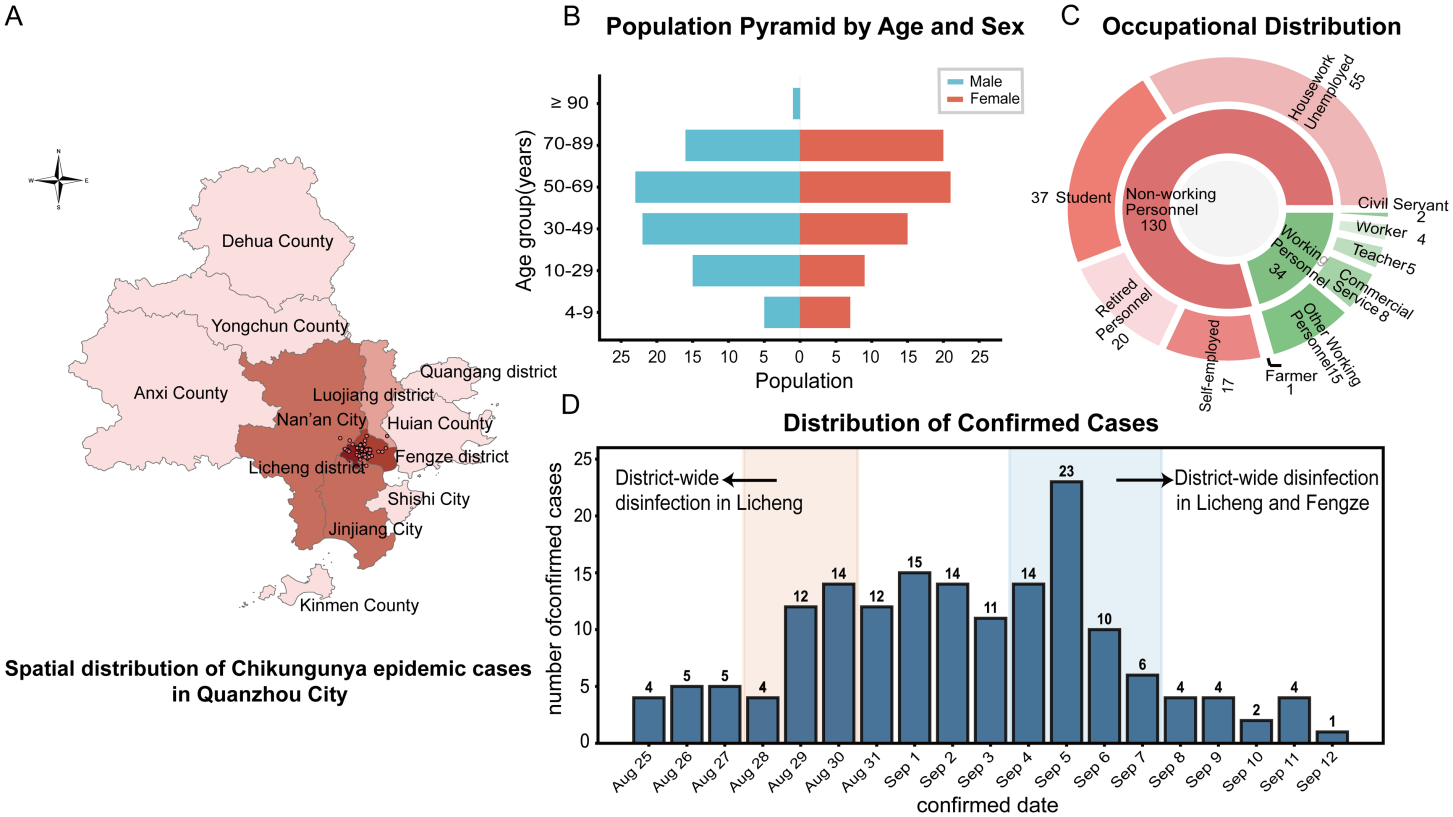
Spatiotemporal and demographic distribution of chikungunya fever cases in Quanzhou, 2025. **(A)** Spatial distribution of cases in Quanzhou City. The darker shading indicates higher case density. **(B)** Age and sex distribution. **(C)** Occupational distribution. Inner ring displays major occupational groups, while the outer ring shows subcategories. **(D)** Temporal distribution of confirmed cases. Shaded areas indicate periods of vector control interventions (light orange and light blue).

A total of 164 chikungunya cases were reported across five districts in Quanzhou, with Licheng and Fengze Districts accounting for 71.3% of all cases (Figure 1A). The male-to-female ratio of cases was 1:1, with an average age of 46.5 ± 24.3 years (median: 49.0 years). Homemakers/unemployed individuals, students, and retirees accounted for 68.3% of all instances (Table 2; Figures 1B,C). The peak reporting period was between August 29 and September 8. Case counts decreased after intensive vector control operations in Licheng District (August 28–30) and both Licheng and Fengze Districts (September 4–7). Daily confirmed cases reduced to less than five after the completion of vector control measures in both districts (Figure 1D).

**Table 2:** Characteristics of Chikungunya cases in Quanzhou, 2025.

Characteristic	n(%)
**Sex**	
Male	82 (50.0)
Female	82 (50.0)
**Age group (years)**	
0 – 9	12(7.3)
10 – 29	34(20.7)
30 – 49	37(22.6)
50 – 69	44(26.8)
70 – 89	36(22.0)
≥ 90	1(0.6)
**Occupation**	
Student	37(22.6)
Teacher	5(3.0)
Commercial/service	8(4.9)
Civil servant	2(1.2)
Office worker	15(9.1)
Manual worker	4(2.4)
Self-employed	17(10.4)
Farmer	1(0.6)
Homemaker/unemployed	55(33.5)
Retiree	20(12.2)

#### 3.1.2 Clinical characteristics

**Figure 2:**
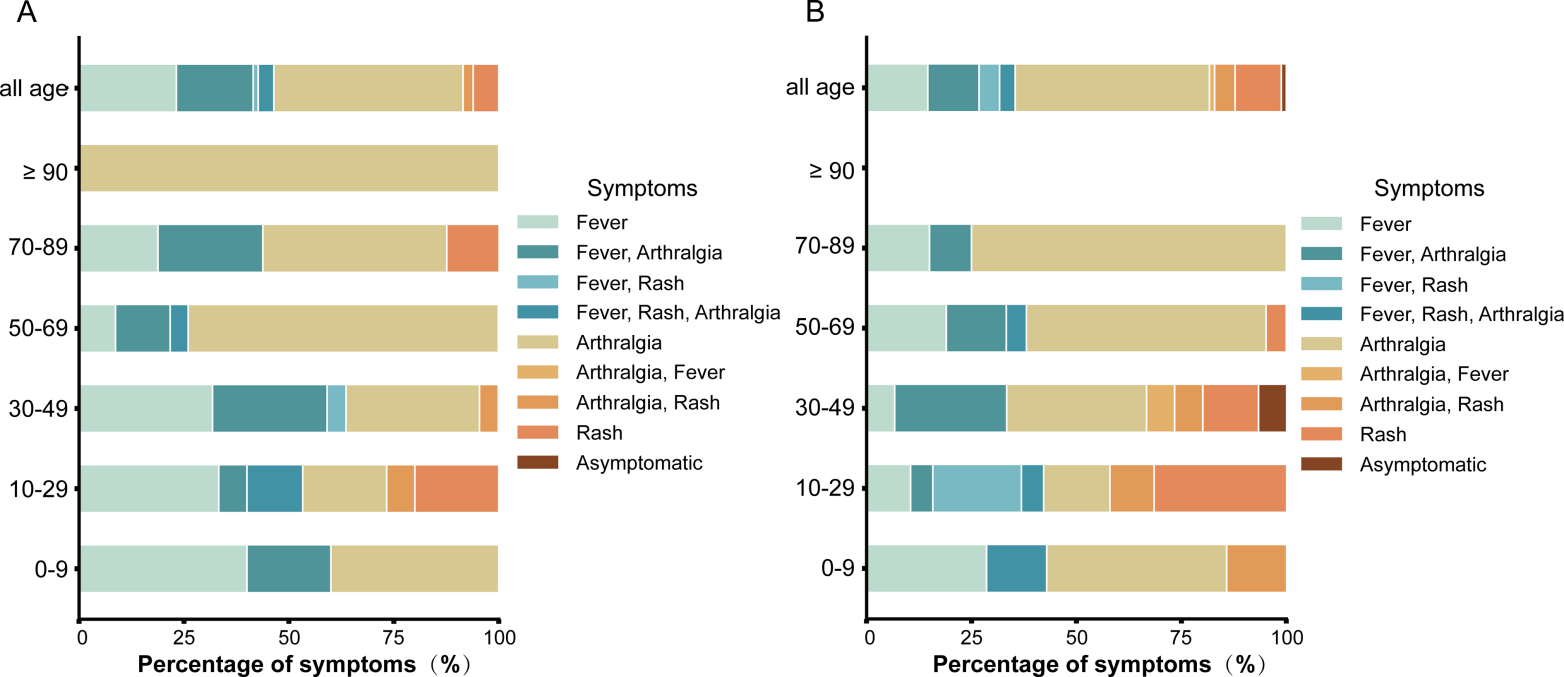
Symptom distribution of Chikungunya fever cases by sex and age in Quanzhou, 2025. **(A)** Stacked bar graph showing the percentage distribution of symptoms by age group in male patients. **(B)** Stacked bar graph showing the percentage distribution of symptoms by age group in female patients.

Joint pain was the most frequently reported symptom among both male and female patients, affecting 45.1 and 46.3%, respectively. Only one female patient (1.2%) was asymptomatic (Figure 2). Statistical testing indicated no significant difference in symptom presentation between genders (*χ^2^* = 8.204, *p* = 0.414). All age groups had a significant prevalence of joint pain except patients aged 10–29 years. The highest occurrence was observed among those aged 50–69 years (65.9%). The single asymptomatic case was a patient in the 30–49 years age group. No statistically significant difference in symptom distribution was found across age groups (*χ^2^* = 0.040, *p* = 0.841).

**Figure 3:**
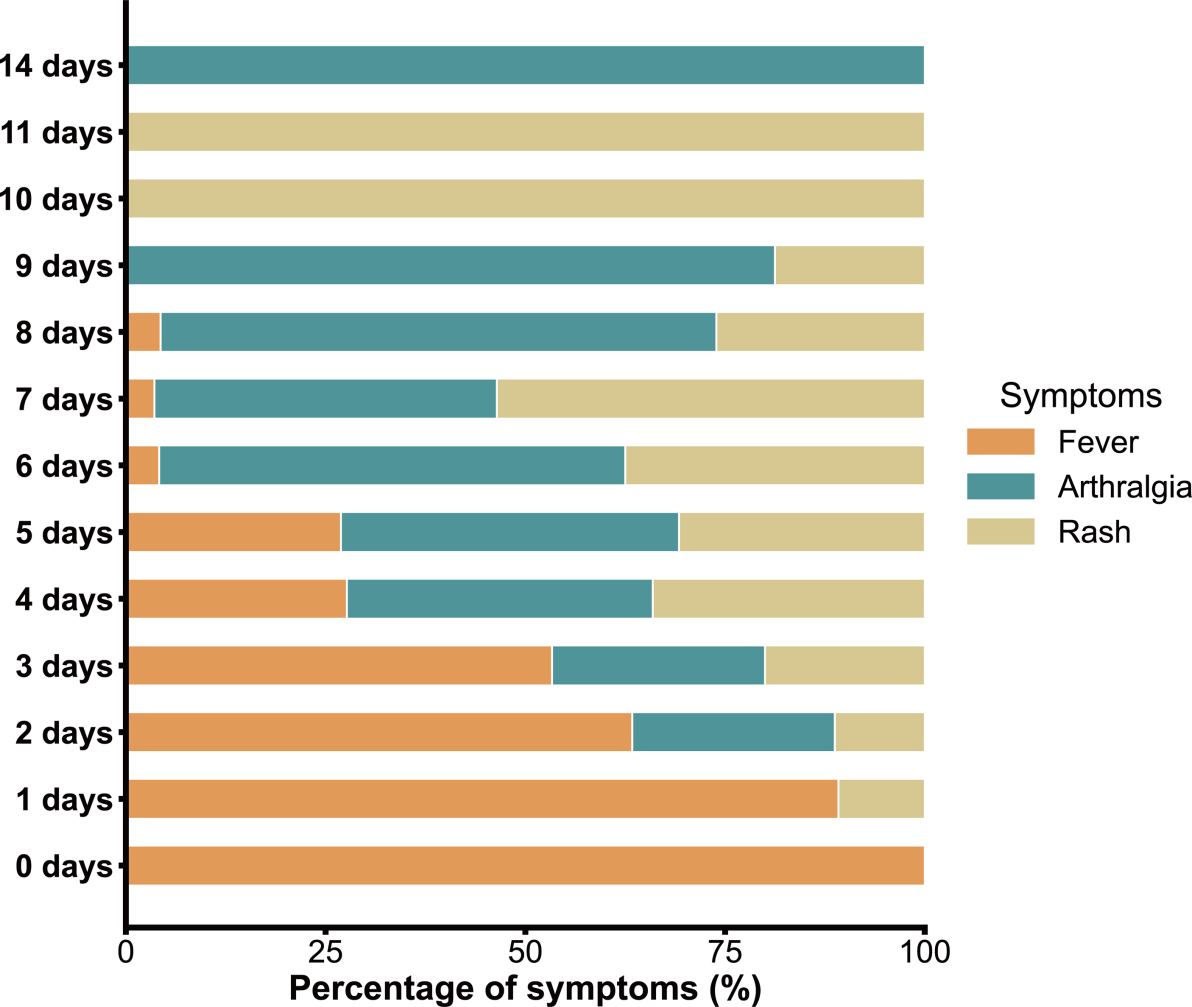
Distribution of Chikungunya fever cases by onset-to-diagnosis interval in Quanzhou, 2025.

Symptom presentation varied according to the time interval between symptom onset and diagnosis. Patients diagnosed within 0–2 days of symptom onset most commonly presented with fever (Figure 3). As the interval extended to 3–5 days, symptom patterns became increasingly diverse with a growing proportion of mixed presentations, notably fever and arthralgia. Beyond this period, symptom composition remained relatively stable.

#### 3.1.3 Differences in demographic characteristics

Chi-square analysis of demographic factors revealed no statistically significant variations in case distribution across age groups (*χ*^2^ = 3.612, *p* = 0.620 > 0.05), occupations (*χ*^2^ = 12.357, *p* = 0.154 > 0.05), regions (*χ* ^2^ = 4.565, *p* = 0.295 > 0.05) and sex (Table 3).

**Table 3:** Demographic characteristics of Chikungunya fever cases in Quanzhou, 2025.

Characteristic	Male (*n*, %)	Female (*n*, %)	*χ* ^2^	*p*
Age group (years)				
0–9	5(6.1)	7(8.5)	3.612	0.620
10–29	15(18.3)	19(23.2)		
30–49	22(26.8)	15(18.3)		
50–69	23(28.0)	21(25.6)		
70–89	16(19.5)	20(24.4)		
≥ 90	1(1.2)	0(0.0)		
Occupation				
Self-employed	12(14.6)	5(6.1)	12.357	0.154
Manual worker	3(3.7)	1(1.2)		
Office worker	10(12.2)	5(6.1)		
Civil servant	2(2.4)	0(0.0)		
Homemaker/unemployed	22(26.8)	33(40.2)		
Teacher	3(3.7)	2(2.4)		
Retiree	8(9.8)	12(14.6)		
Farmer	1(1.2)	0(0.0)		
Commercial/service	5(6.1)	3(3.7)		
Student	16(19.5)	21(25.6)		
District				
Licheng	58(70.7)	58(70.7)	4.565	0.295
Luojiang	1(1.2)	0(0.0)		
Fengze	18(22.0)	23 (28.0)		
Jinjiang	3(3.7)	0(0.0)		
Nan’an	2(2.4)	1(1.2)		

### 3.2 Onset-to-diagnosis characteristics

**Figure 4:**
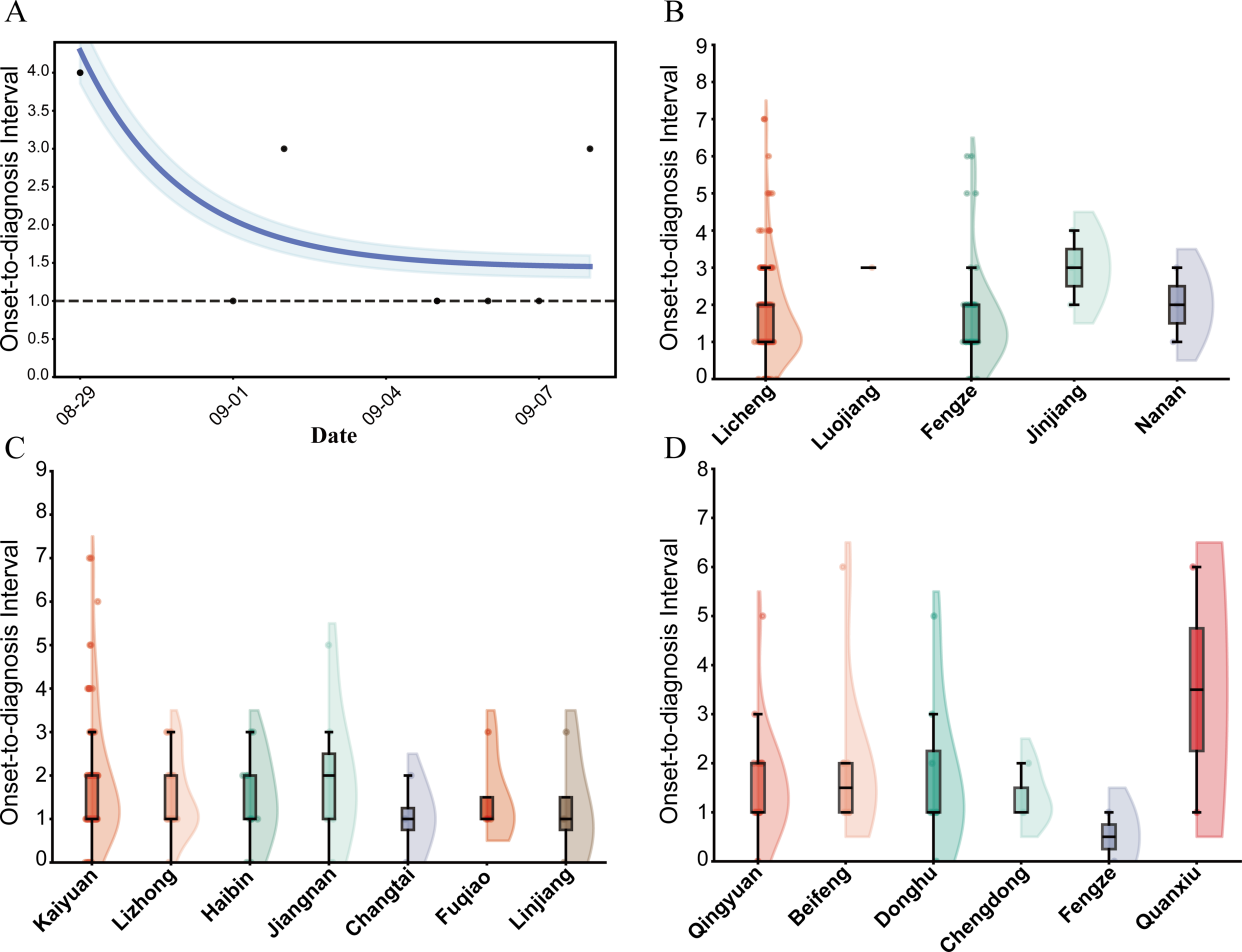
Distribution of onset-to-diagnosis intervals for Chikungunya fever cases by district, Quanzhou, 2025. **(A)** Exponential decay curve of onset-to-diagnosis intervals over time. The blue line represents the trend in onset-to-diagnosis intervals, and the light blue shaded area represents the 95% confidence interval. **(B)** Comparison of onset-to-diagnosis interval distributions across districts of Quanzhou (Licheng, Luojiang, Fengze, Jinjiang, and Nan’an). Box plots show median, IQR and data range. Colored transparent areas represent kernel density estimation distributions, and scatter points show individual data values. **(C)** Distribution of onset-to-diagnosis intervals across subdistricts of Licheng District. **(D)** Distribution of onset-to-diagnosis intervals across subdistricts of Fengze District.

The symptom onset-to-diagnosis interval decreased progressively throughout the outbreak period (Figure 4A). Though some intervals extended to 6–7 days, most patients were diagnosed within 0–2 days of symptom onset. Jinjiang City experiencing notably longer diagnostic delays than other districts, reaching a median interval of 3 days. In contrast, both Licheng and Fengze districts achieved shorter median intervals of 1 day (Figure 4B).

Most chikungunya fever cases occurred in Licheng and Fengze districts. Within Licheng District, Kaiyuan, Lizhong, Changtai, Fuqiao, and Linjiang streets maintained consistent median intervals of approximately 1 day. Despite sharing the same median interval, Haibin Street recorded several outlier cases with periods of 4–7 days or longer. Jiangnan Street exhibited the longest median interval at 2 days (Figure 4C).

In Fengze District, Fengze Street achieved a median diagnostic interval of nearly 0 days, while Chengdong Street maintained a stable median of 1 day. Beifeng Street recorded the most extreme case with a 6-day delay, whereas both Qingyuan and Donghu streets documented cases with delays extending to 5 days. Quanxiu Street exhibited the longest median interval within Fengze District at 3.5 days (IQR: 2–5 days; Figure 4D).

### 3.3 Ct values and infection risk

#### 3.3.1 Univariate analysis of age, onset-to-diagnosis interval, symptoms and Ct values

**Figure 5:**
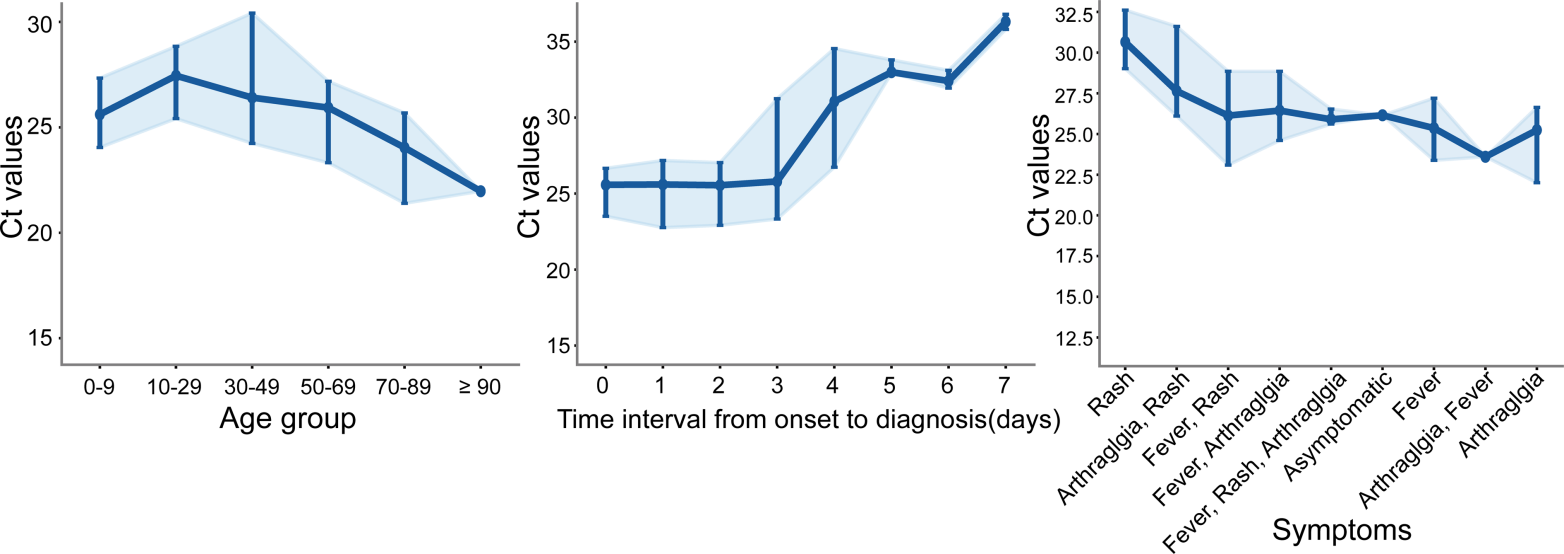
Distribution of Ct values of Chikungunya fever cases in Quanzhou, 2025. **(A)** Trends in Ct values across different age groups. The dark blue solid line represents the mean Ct value for each group, and the error bars and blue shaded area indicate the 95% confidence interval. **(B)** Trends in Ct values across different onset-to-diagnosis interval groups. **(C)** Trends in Ct values across different symptom groups.

Cases showed a statistically significant decreasing trend in Ct values with increasing age (Kruskal-Wallis test, *χ*^2^ = 16.475, *p* = 0.006). Dunn’s post-hoc test with Bonferroni correction revealed significant differences in Ct values between the 10–29 years and 70–89 years age groups (*p* = 0.007), as well as between the 30–49 years and 70–89 years age groups (*p* = 0.018; Figure 5A).

The relationship between Ct values and onset-to-diagnosis interval is presented in Figure 5B. Ct values remained below 30 during the first 3 days following symptom onset, after which the median values increased sharply and progressively (Kruskal-Wallis test, *χ^2^* = 27.311, *p* = 0.001). Dunn’s post-hoc test with Bonferroni correction found significant changes in Ct values between groups at 1 and 5 days (*p* = 0.031) and 2 and 5 days (*p* = 0.049).

Most individuals with common symptoms such as fever, arthralgia, or both, had Ct values in the comparatively low range (23–27). Patients with arthralgia and rash or rash had significantly higher median Ct values of around 30. Asymptomatic individuals demonstrated Ct values comparable to those with other symptoms. Overall, Ct values differed significantly across symptom combinations (Kruskal-Wallis test, *χ*^2^ = 25.823, *p* = 0.001). Dunn’s post-hoc test with Bonferroni correction identified a significant difference in Ct values between the arthralgia and rash groups (*p* = 0.046; Figure 5C).

**Figure 6:**
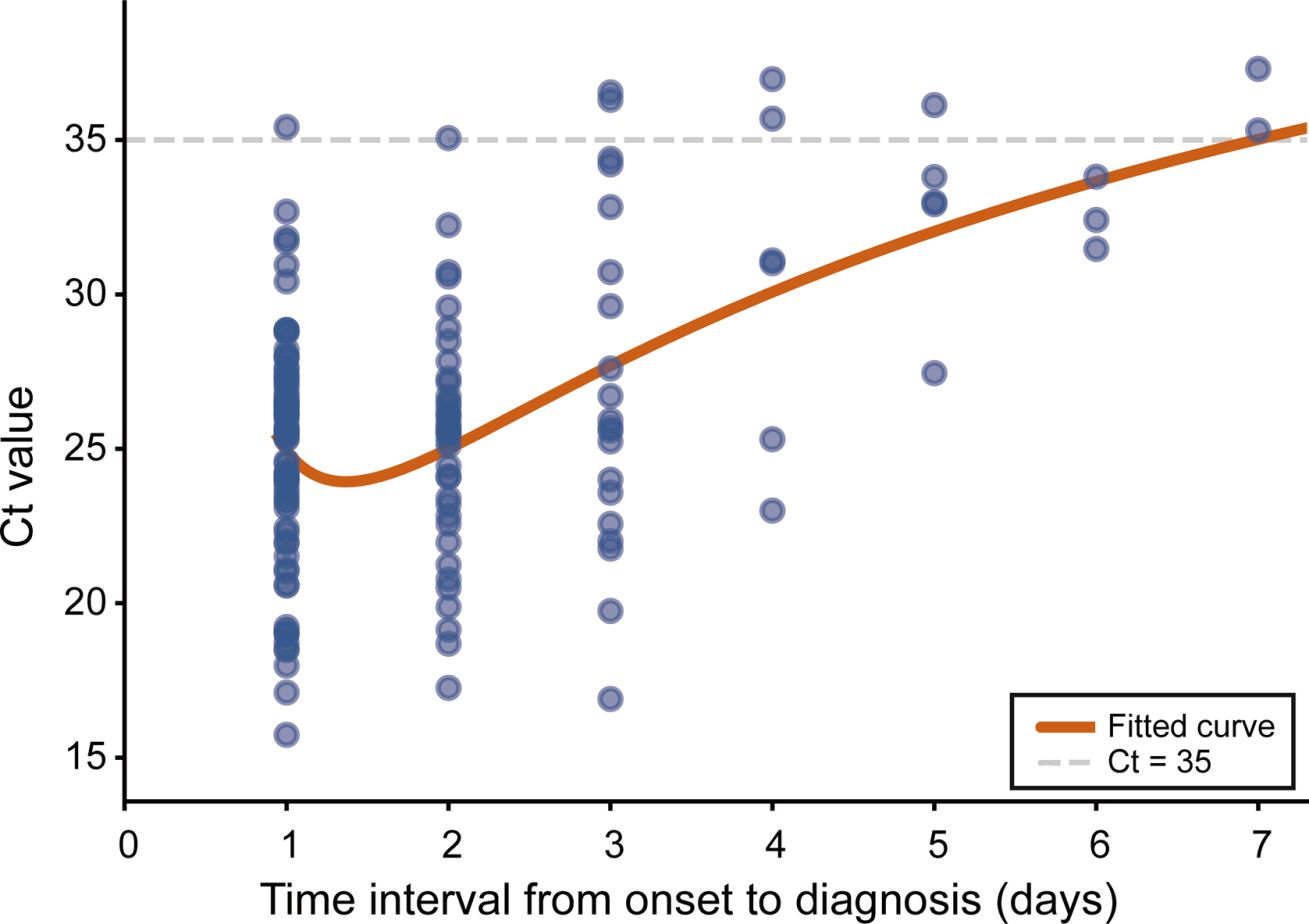
Trends in Ct values by onset-to-diagnosis interval.

Fitting case Ct values to the onset-to-diagnosis interval resulted in the functional expression $y=59.53-83.22x^{-0.5}+48.65x^{-1}$ . All three fitted parameters (b_1_, b_2_, b_3_) were statistically significant (*P* < 0.01). Ct values were lowest in the early stage of illness, reaching a minimum value of 15 near symptom onset, and subsequently increased to peak at day 4 (Figure 6). This aligns with the previously observed distribution.

#### 3.3.2 Multivariate analysis of factors associated with Ct values

Multiple linear regression analysis identified age (*p* = 0.0044) and onset-to-diagnosis interval (*p* < 0.0001) as the only significant predictors of Ct values. Both variables exhibited positive correlations with Ct values, indicating that viral load decreased with increasing age and with longer onset-to-diagnosis intervals (Table 4).

**Table 4:** Factors associated with Ct values.

Variable	Partial regression coefficient	Standard error	Standardized regression coefficient	*t*	*p*	*R^2^*	*Adjusted R^2^*
Sex	0.093	-	0.010	0.146	0.8840	0.2540	0.2255
Age	−0.811	0.281	−0.225	−2.887	0.0044		
Occupation	0.098	-	0.063	0.801	0.4242		
District	0.102	-	0.023	0.335	0.7382		
Onset-to-diagnosis interval	1.412	0.229	0.434	6.176	<0.0001		
Symptoms	0.100	-	0.048	0.680	0.4977		

#### 3.3.3 Factors influencing individual infection risk

**Figure 7:**
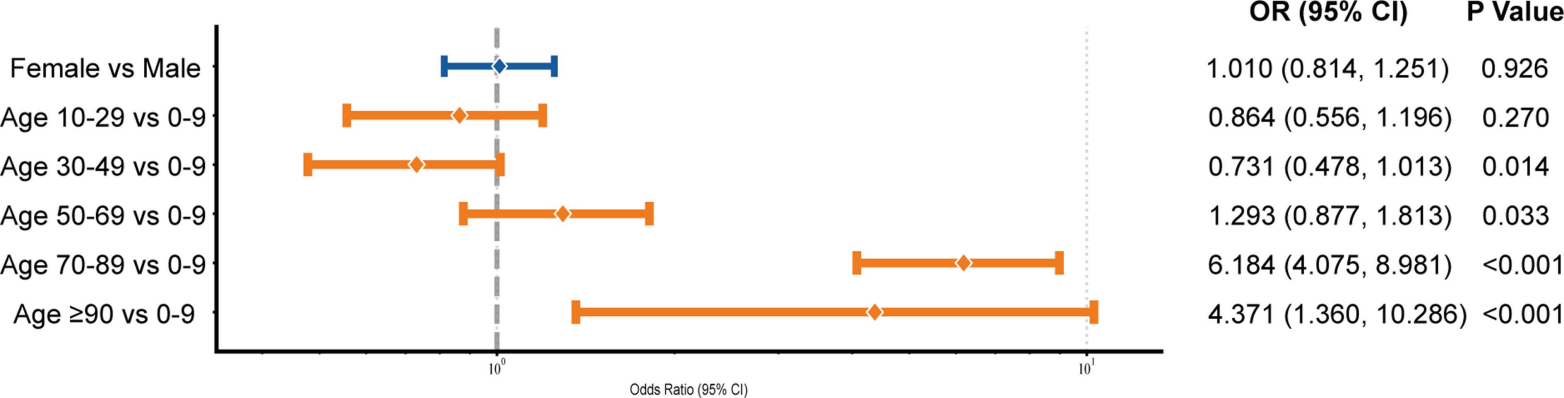
Association between demographic factors (sex and age) and individual infection risk.

The association between demographic characteristics and infection risk was evaluated for sex and age. Age emerged as a main risk factor for chikungunya fever infection (70–89 years group: OR = 6.18, 95%CI = 4.08–8.98; ≥ 90 years group: OR = 4.37, 95%CI = 1.36–10.29). Conversely, sex was not significantly associated with infection risk (Figure 7).

### 3.4 Impact of vectors on disease transmission

#### 3.4.1 BI distribution

**Figure 8:**
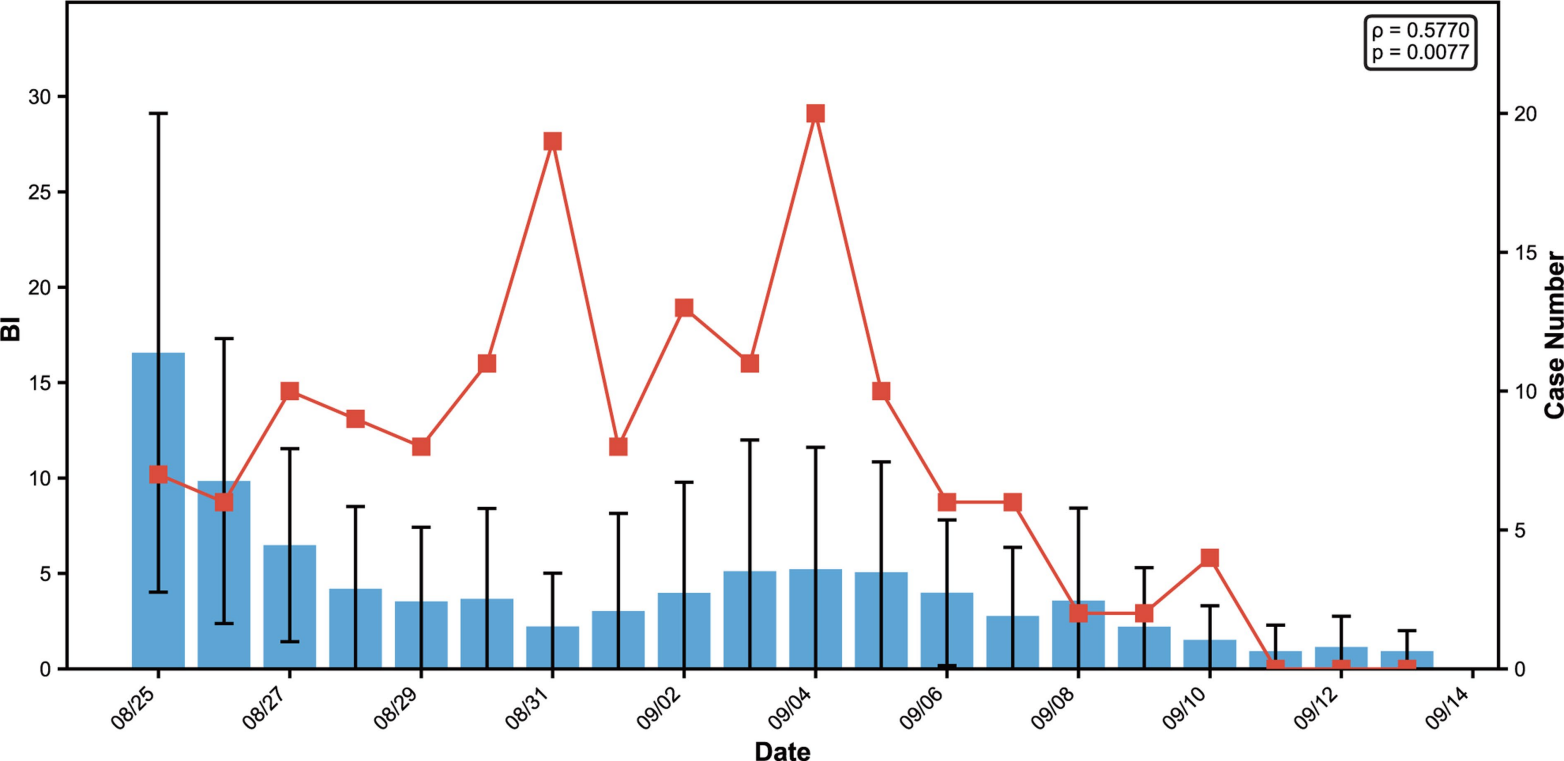
Distribution of BI of Chikungunya Fever cases in Quanzhou, 2025.

BI followed a rise-and-fall pattern throughout the outbreak period (Figure 8). Two daily case counts peaked on August 31 and September 4, reaching a maximum of nearly 30 cases. Correlation analysis revealed a statistically significant positive association between BI and daily case numbers (*ρ* = 0.5770, *p* = 0.0077 < 0.05).

#### 3.4.2 ADI distribution

**Figure 9:**
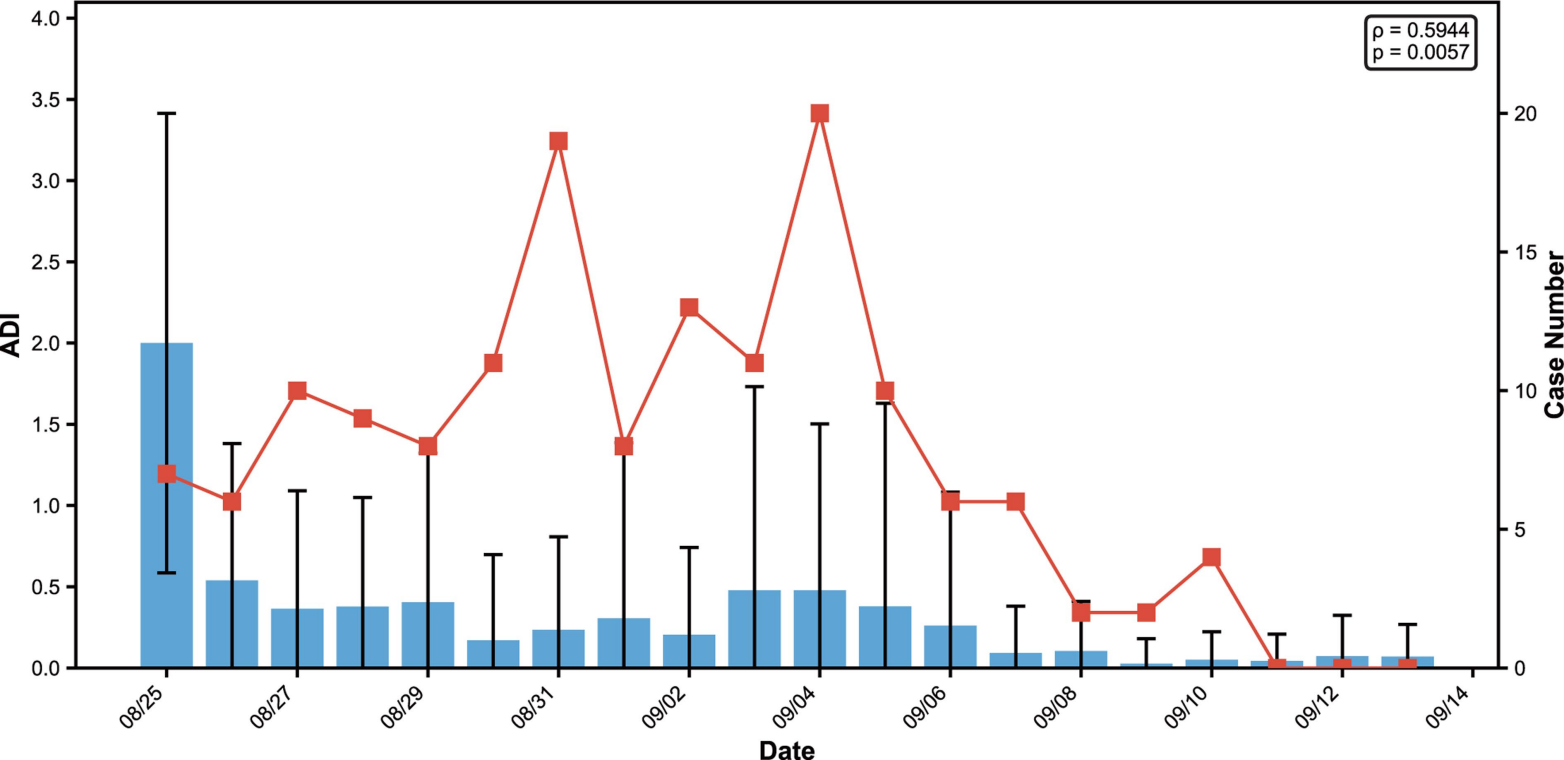
Distribution of ADI of Chikungunya fever cases in Quanzhou, 2025.

The overall trend of ADI paralleled that of BI throughout the outbreak period (Figure 9). Correlation analysis found a significant positive association between ADI and daily case numbers (*ρ* = 0.5944, *p* = 0.0057 < 0.05).

#### 3.4.3 BI and ADI in relation to infection risk

**Figure 10:**
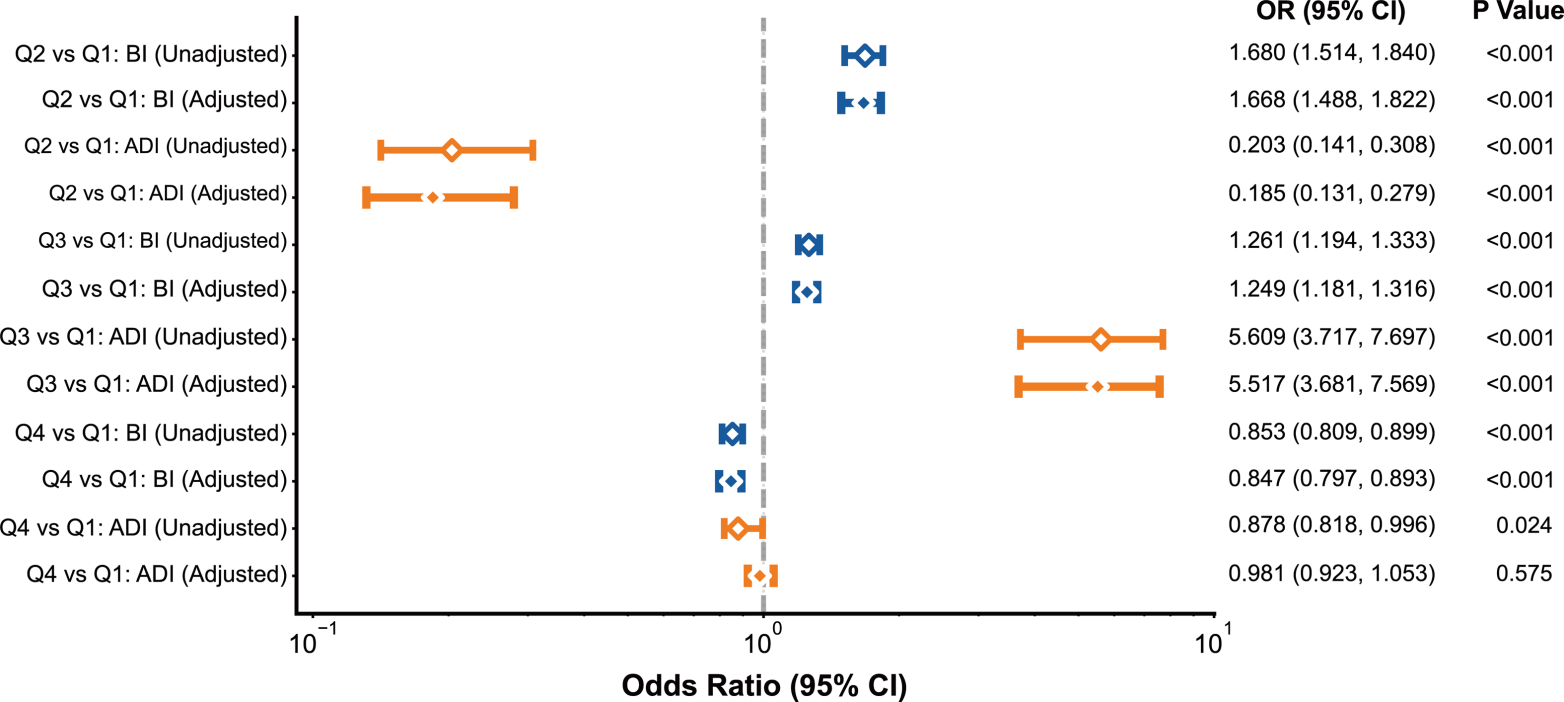
Correlation analysis between BI, ADI and daily incidence levels. Daily case counts were categorized into four groups (Q1 to Q4) based on the 25th percentile, median, and 75th percentile, with Q1 serving as the reference group.

The association between infection risk and both BI and ADI were investigated. Both BI (Q2: OR = 1.68, 95%CI = 1.51–1.84; Q3: OR = 1.26, 95%CI = 1.19–1.34) and ADI (Q3: OR = 5.60, 95%CI = 3.74–7.70) were identified as significant risk factors for chikungunya fever infection. The results persisted after adjusting for potential confounding variables including age and sex (Figure 10).

## 4 Discussion

Based on data from the 2025 chikungunya outbreak in Quanzhou, Fujian Province, China, this study detailed and examined the epidemiological characteristics, symptom distribution, Ct value trends, and vector patterns of the outbreak. During the outbreak, interventions including case isolation and treatment, vector control, and emergency response effectively reduced daily case incidence, as supported by corresponding declines in mosquito surveillance indicators.

Currently, chikungunya fever has not established endemic transmission in China, with cases mainly consisting of imported infections and associated risk of local spread. Following the detection of an imported case in Shunde District, Foshan City, Guangdong Province, a total of 4,754 confirmed cases were reported through July 26, corresponding to an incidence rate of 49.44 per 100,000 population (20). While the Quanzhou outbreak shared epidemiological similarities with the Foshan outbreak, the two differed in case burden. Several factors account for this variation. First, Fujian Province reported fewer imported cases than Guangdong Province. As a major center for foreign trade and a primary destination for Southeast Asian labor migration, Guangdong faces elevated risks associated with population mobility and international case importation (21). The Foshan outbreak showed distinct clustering patterns related to age, occupation, and household transmission (22). While the Quanzhou outbreak demonstrated some occupational clustering with homemakers and unemployed individuals accounting for 33.5% of cases, other clustering patterns were not observed. Delayed case detection and diagnosis have been identified as critical factors driving community transmission and regional spread of chikungunya fever (23, 24). Fujian Province conducts routine vector surveillance and imported case management for mosquito-borne diseases, immediately implementing health alerts and personnel measures following outbreak emergence in neighboring provinces (21, 25). After activation of the emergency response on August 25, Quanzhou rapidly deployed case isolation and vector control interventions, effectively limiting local transmission risk.

Analysis of Ct value distribution discovered that viral load increased gradually with advancing age, consistent with previous studies suggesting an association between older age and increased viral loads (26, 27). High viral loads were detected in patients immediately following symptom onset and declined after day 3 post-onset. The *Diagnosis of Chikungunya Fever* (WS/T 590–2018) (28), and studies from other endemic regions (29–31) support this temporal pattern, which indicate that chikungunya fever is characterized by peak viremia during days 1–2 after symptom onset, with subsequent decline on days 3–4. Patients with fever, fever and arthralgia, or other combinations of these symptoms exhibited relatively low Ct values. This result has been confirmed in recent studies on clinical characteristics and viral loads of chikungunya cases, suggesting that fever and arthralgia represent the most characteristic symptom complex associated with high viral load (32–34). However, symptoms did not emerge as a significant predictor of Ct values in multivariate analysis. Based on these results, infectiousness may remain comparable across asymptomatic, symptomatic, and severe cases. Limited evidence currently supports this hypothesis (35, 36) and further investigation is needed.

Infection risk varies by age, with individuals aged 70 years and above being at highest risk. Previous studies and official guidelines identify individuals aged 60 or 65 years and above as high-risk populations for chikungunya fever, broadly consistent with our findings (10, 37, 38). Deviating from a monotonically increasing trend, the OR for the ≥ 90 years age group was notably lower. This pattern is likely attributable to instability in the effect estimate, as just one case was reported in this age group (95% CI: 1.36–10.29). Reduced outdoor activity leading to lower mosquito exposure, as well as the potential healthy survivor effect among those reaching age 90 and above may also contribute to this attenuation. Timely intervention measures demonstrated clear effectiveness. The epidemic curve showed a marked decline following intervention implementation, with all vector surveillance indicators decreasing to levels within acceptable control thresholds. This conclusion is consistent with outcomes observed during the 2019 imported outbreak in Ruili, Yunnan (39) and the 2025 outbreak in Foshan, Guangdong (22), emphasizing the importance of timely case detection and isolation combined with mosquito surveillance and control.

This study has several limitations. As only one asymptomatic case was identified among the 164 cases included in this study, the conclusion that asymptomatic patients may be as infectious as symptomatic cases is preliminary and should be validated in future studies with larger sample sizes. Potential confounding factors such as comorbidities, prior flavivirus or alphavirus exposure, and housing and socioeconomic situations (e.g., air conditioning, housing type) were not considered, which may have influenced the estimated associations between infection risk and age or occupation. Incorporating these variables with appropriate statistical adjustment for prior exposure would further improve the accuracy of risk estimates in subsequent studies. Comparable studies have indicated that reductions in vector indices following targeted interventions correspond with decreasing case trends during outbreaks (40, 41). However, as case isolation, health alerts, and vector control measures were implemented within a short time in this study, it is difficult to attribute the outbreak’s decline solely to any single intervention. Future investigations employing prospective study designs or calibrated transmission dynamic models would enable more precise attribution of effects to particular interventions.

## Data Availability

The original contributions presented in the study are included in the article/supplementary material, further inquiries can be directed to the corresponding authors.
